# Evaluating public and patient involvement in interventional research–A newly developed checklist (EPPIIC)

**DOI:** 10.1371/journal.pone.0301314

**Published:** 2024-11-05

**Authors:** Elise Pyne, Robert Joyce, Christopher P. Dwyer, Sinéad M. Hynes

**Affiliations:** 1 School of Medicine, College of Medicine, Nursing and Health Sciences, University of Galway, Galway, Ireland; 2 School of Health Sciences, College of Medicine, Nursing and Health Sciences, University of Galway, Galway, Ireland; 3 HEA Performance & Dept. of Teacher Education, Technological University of the Shannon, Athlone, Ireland; University of Toronto, CANADA

## Abstract

Public and patient involvement (PPI) has been identified as an increasingly desired and, often, required component of trial methodology–leading to higher quality, more accessible and relevant clinical research, alongside increased recruitment, funding success and insight into research impact. However, despite the great variety of frameworks and checklists available for assessing PPI, most are limited with respect to important features (e.g. applicable in specific contexts only, fail to clarify what should be assessed and reported, lack the necessary comprehensiveness or are biased in favour of researcher reporting). Thus, the current research aimed to address such limitations through the development of a new checklist, the EPPIIC, through review, thematic analysis and ‘meta-evaluation’ in conjunction with PPI engagement. Upon completion of the EPPIIC, three thematic ‘sub-scales’ emerged: (1) Policy & Practice, (2) Participatory Culture and (3) Influence & Impact. All findings are presented and discussed in light of theory and research. Notably, findings recommend EPPIIC as a useful means of assessing PPI in future trials.

## Introduction

Public and patient involvement (PPI) is vital for trial methodology, leading to higher quality, more accessible and relevant clinical research [1]. It is also rapidly becoming a more-and-more desired–if not required–component of clinical trials [2]. Recent approaches to PPI aim to empower and enable such member involvement, allowing for flexible structures and procedures created by both PPI members and researchers. ‘*Nothing about us without us’*–a message often used by PPI members in context–tells clinical researchers that the raw purpose of their work is to improve the lives of those affected by the topic of their study [3]. While researchers may understand the intricate pathology of disease, it is patients who have the unique lived experience of the condition.

PPI inclusion further increases recruitment of study participants, funding success, and facilitates unique insights for discussion regarding potential impacts of the research [4]. For PPI members, involvement can increase skills and boost feelings of self-worth and confidence [5]. Notably, *true* PPI extends beyond mere consultation to active partnership throughout the research’s life-cycle–from PPI involvement in funding applications and protocol development all the way to dissemination and knowledge translation [6]. Indeed, through appropriate implementation, PPI members can be the invaluable ‘critical friends’ needed to improve the overall quality of clinical research [7, 8].

With pressure from funders to embed PPI into clinical research and improved awareness of the benefits of PPI, the rate of PPI in clinical research has rapidly increased [2, 9]. However, the evidence of PPI impact is less clear, with continued discussion and debate concerning the means of evaluating the use of PPI in clinical trials [10, 11]. Though there is vast agreement regarding the need to capture the negative and positive aspects of PPI processes [10], there exists a variety of frameworks, surveys and checklists–with diverse perspectives that claim to capture the challenges faced and opportunities created when using PPI in clinical research [12, 13]. However, the comprehensiveness and focuses of these tools are debatable in light of this diverse pool from which to choose–debate further reinforced by the relative recency of PPI as a phenomenon in clinical trial methodology. Not surprisingly, there also exists a demand for a guideline and/or framework that not only evaluates PPI, but also provides researchers with clarity regarding what *should be* assessed and reported, in context [12]. Again, ‘context is key’ and, unfortunately, not all PPI evaluation strategies are contextually appropriate, when such comprehensiveness is desired. Indeed, comparison and appraisal of strategies, assessing impact and ensuring what is claimed has been done, are at the heart of evaluating PPI approaches.

None of these extant PPI checklists are without their limitations [13]. Most frameworks evaluate from the researcher’s perspective–a strategy that immediately suggests reporting bias. On the other hand, evaluation strategies that do account for PPI members responding are also problematic, with one review finding that only 11.1% of tools had the reading level sufficient for public or lay persons’ understanding [11]. Many of these also fail to address the same areas from both perspectives- the PPI member(s) and the researcher(s). Typically, PPI members are only questioned on their input rather than the accommodations that have been made for them by the researcher team, whereas researchers have the opportunity to comment on both.

Thus, the focus of the current research is to address the limitations of previous evaluation tools through the development of a new checklist that includes both researcher and PPI member forms for comparative evaluation. The checklist aims to be generalisable across research typologies within the parameters of clinical interventions. The checklist will allow for a comprehensive description of PPI focuses, though non-specific reporting cues with added focus on open-ended reporting. This has been achieved through 1) a comprehensive and critical review of current PPI guidelines and outcome measures; 2) collation of thematically identified areas to create an evaluation checklist (or checklists) to appraise the quality of PPI within trials; 3) and initial piloting and application of the checklists (available in S3 Appendix: Application of the EPPIC to the COB-MS feasibility trial, using a feasibility trial as an example [14, 15]).

## Methods and findings

### Review of current tools

A literature review of extant PPI checklists was conducted, focusing on the process and outcome assessments of PPI. These checklists were then subjected to content analysis to identify common topics throughout and identify the exact quantitative and qualitative questions and methods to evaluate PPI. This work was completed in consultation with PPI member (RJ). Variations in the style of existing checklists also provided insight in the best way to formulate questions.

### Search strategy

To navigate and formulate the research question, the SPIDER search strategy was used [16] (see Table 1) to ensure the relevant and appropriate frameworks were being evaluated. Other search methods included a keywords search using PubMed and Google Scholar search engines. Boolean operators were used to carry out an extensive search of all related research and documentation (see Table 2).

**Table 1:** Identification of research question through SPIDER Search strategy.

S Sample	PI Phenomenon of Interest	D Design	E Evaluation	R Research Type
Various PPI evaluation frameworks used in clinical trials	Efficacy of framework to report PPI	Using PPI Evaluation frameworks and modifying them to fit this trial	Impact of PPI on study recruitment, retention, and overall trial quality	Mixed methods

**Table 2:** Keywords used in Boolean operators to identify research concerning PPI evaluation strategies.

Public and Patient Involvement OR PPI OR Patient Engagement OR Patient Participation OR User Involvement	AND	Framework OR checklist OR criteria OR agenda OR Method OR Approach OR Guideline OR model OR Toolkit OR Strategy	AND	Evaluation OR Assessment OR Appraisal

In addition to the literature review, the Centre of Excellence for Partnership with Patients and the Public (CEPPP) database was also used to identify relevant checklists for this research. The CEPPP is an online resource that encompasses a number of evaluation tools, to enable researchers to assess the quality of PPI in their research. The CEPPP evaluates the included tools based on usability, comprehensiveness, patient and public perspective and scientific rigour by applying targeted questions to each framework. From CEPPP’s previous evaluation, each framework was investigated, with 11 satisfying our inclusion criteria. From the continued literature review, a further nine checklists were examined and satisfied the inclusion criteria. An overview of the contents of each checklist is included in Table 3 below.

**Table 3:** Summary overview of checklists satisfying inclusion criteria and utilised in reviewing themes.

No.	Checklist	Overview of contents
**CEPPP Checklists**		
1.	Engagement Toolkit,—*H*.*P*.*O*.*I*.*H*. *System*, *2016* [22]	Checklist including all areas of PPI best practice in addition to questionnaire to analyse team collaboration skills.
2.	PPEET—*Public and Patient Engagement Collaborative MU* [20]	Set of questionnaires to gather opinions and experiences of PPI members for short-term and long-term participation.
3.	Rifkin Spidergram—*Rifkin SB*, *et al*., *1988* [25]	Use of a plotting system based on 5 separate characteristics of PPI to visually compare and contrast advances and shortcomings between trials.
4.	STEPP—*Kreindler SA et al*., *2016* [38]	Checklist designed to monitor and retrospectively review PPI within trials by using a score sheet format
5.	The Participation Toolkit, *Scottish health Council 2014* [34]	Toolkit and questionnaire designed to evaluate facilitation of public and patient members and to assess planning.
6.	Well connected–a self-assessment tool on community involvement *South J*, *et al*., *2005* [27]	This tool focused on a scoring system to analyse public involvement in research with the aim of identifying strengths and weaknesses from this involvement.
7.	An Evaluation of In-Person and Online Engagement in Central Newfoundland *Wilton P*, *et al*., *2015* [30]	Assessing in-person and virtual collaboration and implementation in a trial in Newfoundland. Results of questionnaire were examined and retrospectively showed need for the checklist.
8.	PAIR *Arora PG*, *et al*., *2015* [31]	Checklist includes 5 ‘dimensions’ for examination of PPI used in trials, with specific goal to comment on collaboration, benefits, and lessons learned.
9.	PiiAF *Group PS*, *2014* [21]	Checklist taking a two-pronged approach to evaluate firstly the structure and initial planning for PPI and secondly methods to evaluate the impact and contribution of PPI.
10.	RAPPORT *Wilson P*, *et al*., *2015* [35]	Reporting of PPI within six trials using a three-stage approach including dividing types of PPI into a ‘one-off’ model, fully intertwined model and an outreach model.
11.	Patients as Partners in Research, *Maybee et al*., *2016*, *Maybee et al*. *2016* [23, 24]	Questionnaire for patient/caregiver and researcher groups to assess quality and organisation of collaboration in PPI.
**Literature Review Yield Checklists**		
1.	The Public and Patient Engagement Evaluation Tool *Garratt A et al*., *2022* [19]	Checklist to evaluate PPI for improve health care services and organisation of these methods.
2.	Checklist for Public Involvement in Clinical Effectiveness Processes *Committee NCE 2018* [26]	Questionnaire based evaluation of PPI used in trials for patient participants including pre-trial deliberations and collaboration throughout trial.
3.	Metrics and Evaluation Tools for Patient Engagement in Healthcare Organisation *Dukhanin V et al*., *2018* [28]	Review of advantages and disadvantages of current PPI evaluation checklists including pointers for future research.
4.	A protocol for the evaluation of the process and impact of embedding formal and experiential Public and Patient Involvement training in a structured PhD programme *Foley L et al*., *2021* [29]	A protocol of using focus groups, reflections and individual interviews to assess PPI use during PhD programmes.
5.	Public Participation Methods *Rowe G*, *Frewer*, *L*. *J*, *2000* [13]	A checklist concerned with theoretically evaluating PPI by analysing acceptance and process criteria.
6.	Evaluating Organisational Collaborations *Woodland RH*, *Hutton*, *M*. *S*., *2018* [32]	An accumulation of 5 aims of PPI evaluation including describing participation, examining collaboration and measure effects of participation over time.
7.	GRIPP2 *Staniszewska S et al*., *2017* [33]	An international approach to evaluating PPI with an evidence-based checklist which aims to evaluate transparency and quality of PPI in research.
8.	Dialogue Model Applied *Broerse JE et al*., *2010* [36]	Application of the dialogue model to a trial identified similarities and differences in the ideas of the research group compared to the patient group.
9.	Dialogue Model *Abma TA*, *Broerse JE*, *2010* [37]	This checklist contained six phases of evaluation of PPI based on review of case studies related to chronic disease management.

### Inclusion / exclusion criteria

The only inclusion criterion for framework evaluation was that the framework must assess PPI in the context of clinical research. Frameworks were excluded if they were not relevant to research or deviated from it -e.g. solely focused on team dynamics and collaboration.

### Analysis of extant frameworks

After all relevant checklists had been identified, thematic analysis was conducted, to identify, analyse and report themes within the qualitative data. Specifically, data were analysed consistent with Braun and Clarke’s [17, 18] six-phase analytic process, which highlights three main tasks: familiarisation with data; coding and theme identification; and the reviewing and refining of themes. This method included reading and re-reading of frameworks to a gain familiarity with the materials, prior to identification of components of interesting elements, codes, and approaches. An extensive list was generated and sorted into overarching themes.

## Results

Through thematic analysis, many overlapping patterns of themes were found. Subthemes were grouped into their overarching theme to create each of the three main themes. A summary of the research evidence that led to identification of these subthemes is presented in Table 4. Table 4 presents the theme and subthemes that were identified (column 1). Column 2 identifies the checklists or resources used to identify these themes and sub-themes. The final column of Table 4 (Item #) maps where these areas are presented in the newly-developed checklists. See S1 Appendix: Evaluation of PPI for Interventional research Checklist (EPPIIC). EPPIIC (PPI Version and S2 Appendix: Evaluation of PPI for Interventional research Checklist (EPPIIC). EPPIIC (Researcher Version) for the checklists and included items.

**Table 4:** Summary of themes and subthemes identified from PPI evaluation tools.

*Theme and Subtheme*	*Checklists*	*Item #*
**Policy & Practice**		
Planned Strategy and Methods	[13, 19–34] Public Participation Methods, The Public and Patient Engagement Evaluation Tool, PPEET, PiiAF, Engagement Toolkit, Patients as Partners in Research, Rifkin Spidergram, Checklist for Public Involvement in Clinical Effectiveness Processes, Well-Connected, Metrics and Evaluation Tools for Patient Engagement in Healthcare Organisation, Foley’s (2021) checklist, PAIR, Woodland’s (2018) checklist, GRIPP2, The Participation Toolkit, Wilton’s (2015) checklist	PPI Form– 1, 2, 3, 4, 5, 6, 7 Researcher form– 1, 2, 3, 4, 5, 6, 7, 8, 9, 10, 11, 12
Resource mobilisation	[13, 19–20, 22–28, 31, 33, 35–37] Public Participation Methods, The Public and Patient Engagement Evaluation, PPEET, Engagement Toolkit, Dialogue Model, Broerse’s (2010) Application of the Dialogue Model, Patients as Partners in Research, Rifkin Spidergram, Checklist for Public Involvement in Clinical Effectiveness Processes, Well-Connected, Metrics and Evaluation Tools for Patient Engagement in Healthcare Organisation, PAIR, GRIPP2, RAPPORT	PPI Form– 13, 14, 15, 16, 17, 18, 47, 48, 49, 50, 51, 52 Researcher Form– 13, 14, 15, 16, 17, 18, 19, 20, 21, 23, 24, 24, 25, 26
Reports of PPI	[13, 19–24, 26, 28, 31, 33–38] Public Participation Methods, The Public and Patient Engagement Evaluation Tool, PPEET, Patients as Partners in Research, PiiAF, Engagement Toolkit, Checklist for Public Involvement in Clinical Effectiveness Processes, Metrics and Evaluation Tools for Patient Engagement in Healthcare Organisation, PAIR, GRIPP2, The Participation Toolkit, RAPPORT, Broerse’s (2010) Application of the Dialogue Model, Dialogue Model, STEPP	PPI Form– 8, 11, 12 Researcher Form– 27, 28
Recruitment	[32, 35] Woodland’ (2018) checklist, RAPPORT	PPI Form– 9, 10 Researcher Form– 29, 30, 31
Team Engagement	[21–25, 28, 31, 32] PiiAF, Engagement Toolkit, Patients as Partners in Research, Rifkin Spidergram, Metrics and Evaluation Tools for Patient Engagement in Healthcare Organisation, PAIR, Woodland’s (2018) checklist	PPI Form– 22, 23, 24, 25, 26 Researcher Form– 32, 33, 34, 35, 36, 37, 38, 39, 40
Adaptability	[13, 19, 20, 22, 28, 31, 36, 37] Public Participation Methods, PPEET, The Public and Patient Engagement Evaluation Tool, Engagement Toolkit, Metrics and Evaluation Tools for Patient Engagement in Healthcare Organisation, PAIR, Broerse’s (2010) Application of the Dialogue Model, Dialogue Model	PPI Form– 27, 28, 29, 30 Researcher Form– 41, 42, 43, 44, 45, 46, 47
Experience and representation	[13, 22–25, 27, 31, 35] Public Participation Methods, Engagement Toolkit, Patients as Partners in Research, Rifkin Spidergram, Well-Connected, PAIR, RAPPORT	PPI Form– 41, 42, 43, 44, 45, 46 Researcher Form– 48, 49, 50, 51, 52, 53, 54, 55, 56, 57, 58, 59
Management, and Implementation of PPI Recommendations	[13, 19–22, 28, 38] Public Participation Methods, The Public and Patient Engagement Evaluation Tool, PPEET, PiiAF, Engagement Toolkit, Metrics and Evaluation Tools for Patient Engagement in Healthcare Organisation, STEPP	PPI Form–N/A Researcher Form– 65, 66, 67
Communication methods	[23–24, 27, 31, 32, 34] Patients as Partners in Research, Well-Connected, PAIR, Woodland’ (2018) checklist, The Participation Toolkit	PPI Form– 31, 32, 33, 34, 35, 36, 37, 38, 39, 40 Researcher Form– 60, 61, 62, 63, 64
**Participatory Culture / Collaboration**		
Boosting Awareness	[19–20, 23, 24, 28] The Public and Patient Engagement Evaluation Tool, PPEET, Patients as Partners in Research, Metrics and Evaluation Tools for Patient Engagement in Healthcare Organisation	PPI Form– 53, 54, 55, 56, 57, 58, 59, 60, 61 Researcher Form– 68, 69, 70, 71, 72, 73, 74, 75
Participatory Feedback	[19, 20, 22–24, 26, 31, 33, 34] The Public and Patient Engagement Evaluation Tool, PPEET, Engagement Toolkit, Patients as Partners in Research, Checklist for Public Involvement in Clinical Effectiveness Processes, PAIR, GRIPP2, The Participation Toolkit	PPI Form– 19, 20, 21, 66 Researcher Form– 76, 77, 78, 79
**Influencing Outcomes of PPI**		
Influencing Outcomes of PPI	[13, 21, 35] Public Participation Methods, PiiAF, RAPPORT	PPI Form– 62, 63, 64, 65, Researcher Form– 80, 81, 82, 83

Three main themes were identified: (1) Policy & Practice, (2) Participatory Culture, and (3) Influence & Impact. *Policy & Practice* focused on the structure and strategy of PPI implementation prior to the research starting, centring on the efficiency and methodological aspects of PPI in research–the justification for which was to assess the preparedness of an organisation to carry out this type of research. This item was included as a measure of meaningful involvement and appropriate consideration of PPI. *Participatory Culture* referred to consideration of factors that could enhance or hinder PPI throughout the project, with focus on the research team’s ability to accommodate PPI and how PPI can be optimally engaged/integrated. Notably, this theme is perhaps the main discussion point for comparing trials that include PPI, focusing on the needs of the PPI members and how to appropriately involve them. Finally, *Influence & Impact* focused largely on outcomes of PPI, ensuring that the experience was beneficial to both researchers and PPI members. It reflects the overall impact of PPI on the research question and exhibits whether the PPI strategies initially proposed were actually implemented. This theme is of particular importance, as it not only reveals the advantage(s) of PPI within the trial being evaluated, but also has the capacity to facilitate recommendations for PPI within future interventions.

From this list of themes, a content analysis was completed to formulate the quantitative and qualitative questions required for a comprehensive checklist. Approaches to PPI were divided into formulative and summative questioning, meaning that while some questions related to the process of PPI, others dealt with the final outcomes instead. Developed questions were reviewed to remove anything that was overlapping, repetitive or not directly relevant to PPI. The checklist was reviewed and refined until finalisation.

**Fig 1:**
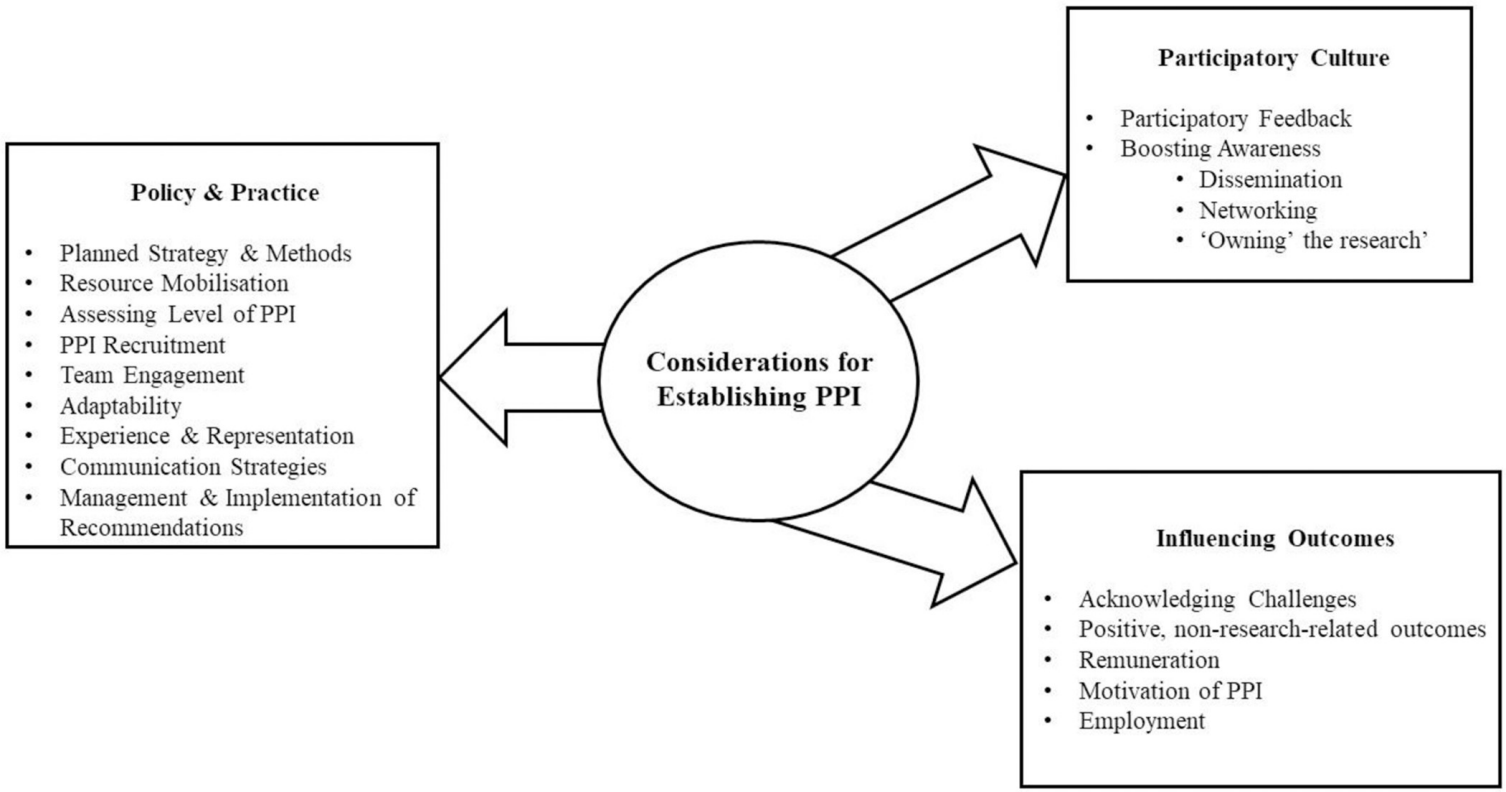
Considerations for establishing PPI, adapted from the EPPIIC.

Relevant questions from extant checklists were collated according to relevant themes and subthemes. After filtering initial questions (e.g. by relevance, overlap and repetition) and amending as appropriate, the resulting questions were organised into two separate forms: researcher evaluation and PPI member evaluation. The final questions were generated by reviewing the wording of questions within other checklists and analysing clarity and comprehensiveness. Some of these were used verbatim, while others were adjusted to ensure better understanding of the question. This process was completed in close collaboration with the research PPI member (RJ). Such organisation reduces bias and facilitates the ability of independent adjudicators to observe perspectives from distinct sources and compare them. Checklist items were tailored to each audience (e.g. with respect to ensuring accessible language) but were otherwise commensurate; though the researcher form includes some additional items (e.g. related to the overall trial budget). Items in the checklists were presented through means of both ‘box-ticking’ (i.e. both Likert scale and dichotomous, yes/no responding, as appropriate) and open-ended response. A balanced mix of formative/summative and qualitative/quantitative inquests were included in both forms. A summary of all steps taken in developing and applying the emerging “Evaluation of PPI for Interventional research Checklist (EPPIIC)” are presented in Table 5 and Fig 1. The finalised EPPIIC checklists can be found in S1 and S2 Appendices. We have also included, in S3 Appendix, application and discussion of the EPPIIC checklists to Cognitive Occupation-Based programme for people with Multiple Sclerosis (COB-MS) [39]. The COB-MS feasibility trial [39] has been used to pilot the checklists and report on the conduct of PPI activities within the trial. Additional discussion has been included in S3 to demonstrate the contextual detail that arises from application of the EPPIIC.

**Table 5:** Summary of steps involved in creation of new EPPIIC.

	Step	Resources/People involved	Results
1	Compile full list of available checklists	CEPPP Database search (PubMed, Google Scholar)	Total checklists identified (n = 20; 11 CEPP and nine database search)
2	Generate full list of themes	All identified checklists; PPI involvement	Total number of items in the first iteration of the checklist = 34
3	Matching each item with a checklist item	All identified checklists	Checklist items were sorted into each identified theme
4	Grouping subthemes into relevant main themes	PPI involvement; research team discussion	Three overarching themes identified
5	Refining the items	PPI involvement; research team discussion	Decision to separate PPI and researcher evaluation
6	Piloting of the checklist	PPI and research team	Adjustments between PPI member and researcher surveys–e.g. change language, merging of items etc.
7	Finalising the checklist	PPI and research team	Number of items–ensuring equal questions between researcher and PPI member survey with appropriate balance of qualitative and quantitative questioning.
8	Application of the checklist	EPPIIC applied to a clinical trial	Results presented below.

## Discussion

The variety of Public and Patient Involvement typologies brings forth great challenge in the development of suitable evaluation tools, applicable across wide-ranging PPI scenarios. Many agree that no individual assessment method can be used [21], which is a barrier to comparing PPI between trials [27]. However, it may be the case that there is a lack of checklists providing sufficient flexibility to assess different PPI contexts. As developed in the current research, the EPPIIC may have the necessary adaptability, as it provides space and opportunities for expression of PPI efforts, in broader contexts, that more specific questioning may miss. While the primary goal of such evaluation exists as an effort to improve PPI standards for future research, identifying tokenistic trials that include PPI just as part of an ‘integrated research agenda’ is also crucial [37]. Although challenging, through consideration of comprehensiveness and attention to PPI specific questioning, it is possible to compare and contrast the strengths and weaknesses of various trials.

Results from our research suggest that the use of open-ended responding at the end of each theme facilitated flexibility in reporting on topics not otherwise addressed.

Notably, a joint qualitative and quantitative approach responding/assessing is seldom used in other checklists but is a necessary component to include for purposes of ensuring a comprehensive evaluation. Qualitative research allows open questioning to assess opinions which allows broader understanding when compared to asking direct quantitative questions [40]. Using open ended questions also allowed for individualised insight within concepts, a much-valued aid in further improvement and assessment, which helps to avoid the ‘tyranny of majority’, in which the generalised opinion dominates individual voices (41), particularly in specific contexts, like those that arise in research interventions This is important in encouraging diverse groups to participate in trials, as facilitating PPI means accommodating to each member’s reality, instead of allowing a needs assessment hospitable to the majority to cover all participants [25].

The EPPIIC, developed in the current research, includes process and outcome metrics, differing from some tools that only consider process-based evaluation [30]. The themes included in the checklist identify the seemingly most vital areas of PPI in research (e.g. practice, culture, and outcomes). Importantly, this checklist can also be used when planning PPI activities (again, see Fig 1).

The largest section of the EPPIIC centres around the structural organisation of PPI within studies, a considerable area of downfall in previous PPI trials [32]. These questions used within the framework pose as surrogates for understanding the factors that led to ‘good’ or ‘bad’ involvement experiences; for example, with respect to training or reimbursement [33]. Some questioning requires an understanding of perspective; for example, participants agreeing that they would participate in the future reflects a positive outlook on their experience [28].

The themes were developed to thoroughly understand the levels of integration of PPI within trials. Often trials adapt the ‘one-off model’ meaning the research team has decided to include PPI for a specific reason, without considering the wider benefits of collaboration [35]. It is often more transactional, with PPI members filling a consultant role rather than as a fellow researcher. Alternatively, the ‘fully intertwined model’ focuses on true participation and PPI member integration within the trial. It reflects the true purpose of PPI and adds the potential to gain all possible benefit, for both the research and the PPI team. Awareness of the approach of integration used reveals the level of consideration and preparation made to include PPI.

The use of two separated perspectives (researcher and PPI member) provided opportunity for divergent focuses which is a vital component of PPI, [28, 41]. Highlighting disparity in opinions between participants is the first step in improving future efforts and reinforces the efficacy of PPI methods. In order to provide their opinions, evaluators must be in an environment of honesty, openness, and respect [31]. Providing separate EPPIICs to both researchers and PPI members facilitated this, as one form was not influenced by the opinions of others. In consideration of the PPI member’s checklist, it was important to adapt the checklist’s language to ensure accessibility and avoid unnecessarily technical jargon and terminology. Other than amendments to language and phrasing, the questions in each checklist remained largely a reflection of each other.

### Limitations and considerations for future research

Another step towards achieving an ideal PPI evaluation in future research would be the use of a possible weighted scoring system for the EPPIIC, where “positive” and “negative” PPI factors could be evaluated within each theme and metricised.

However, as not all questions might receive the same weighting, as one factor could be deemed more crucial to PPI than another, a weighted system would have to be decided within a cohort of PPI members and researchers, which was not possible to carry out in this study. Another challenge of implementing a scoring system is the decision of what would constitute ‘positive’ and ‘negative’ PPI impacts [13], as this is likely to vary considerably across trials/research settings. An example of such a scoring/weighting system is used by the STEPP guideline [38], in which PPI inspired changes to the study are valued as a positive impact. However, in reality, non-implementation of such changes is not always a bad thing. The PPI that is shown in this instance is the ability of the research and PPI team to collaborate and discuss the issue and why a proposed solution is not possible, or maladaptive to the research. Therefore, the labelling of right and wrong needs to be carefully considered. It might also be suggested that such a weighting system might be arbitrary in the light of the potential richness that can be achieved through qualitative responding, as is the case for the current checklist.

Another area for future research would be the an evaluation of PPI throughout a trial (e.g. once every three or six months, perhaps relative to the research’s life-cycle), rather than just at the end [31]. This would allow for presentation of an opportunity to compare the final assessment to understand the impact of continuous evaluation. This feedback could guide PPI efforts to engage more efficient methods within the trial and make amendments, as necessary, in real time. Short assessments following meetings evaluating the effectiveness of communication and involvement aid the effort to improve future meetings and collaboration. Such an evaluation could also give all team members an opportunity to express any issues or queries anonymously, so that their concerns may be discussed at subsequent meetings.

## Conclusion

The EPPIIC, as developed in the current research, expressed a constructivist paradigm that focuses on reflection and notation for future improvement rather than a cynical criticism of possible shortcomings. Having both PPI and Researcher forms of the EPPIIC available allowed for flexibility to evaluate any interventional study using PPI methods. It is hoped this tool leads to further improvement within PPI methods to facilitate use in increasing numbers of clinical trials and studies that will pave the way for optimal research and clinical developments in the years to come.

Overall, the separate EPPIICs allows people to express their thoughts and opinions regarding components of PPI. By comparing perspectives, both strengths and weaknesses of PPI can be highlighted. The findings from this research suggests potential for the EPPIIC’s use in future research, as appropriate. We invite researchers and PPI members alike to use the checklist as a means of evaluating PPI within their own research.

## Supporting information

S1 Appendix Evaluation of PPI for Interventional research checklist (EPPIIC). EPPIIC (PPI Version).
(DOCX)

S2 Appendix Evaluation of PPI for Interventional research checklist (EPPIIC). EPPIIC (Researcher Version).
(DOCX)

S3 Appendix Application of the EPPIC to the COB-MS feasibility trial. (DOCX)
