# Supplementary material for: Evaluating public and patient involvement in interventional research–A newly developed checklist (EPPIIC)
Source: PLoS One. 2024 Nov 5;19(11):e0301314. doi: 10.1371/journal.pone.0301314 (PMC11537402; doi:10.1371/journal.pone.0301314)
Supplement: S1 Appendix — EPPIIC (PPI Version). (DOCX) [file pone.0301314.s001.docx]

**S1:  Evaluation of PPI for Interventional research Checklist (EPPIIC)**

**EPPIIC (PPI Version)**

**Strategy and Methods**

| Was the reason for your PPI engagement communicated to you before starting the project? | | No                  Unsure                   Yes  ☐     ☐     ☐     ☐     ☐     ☐     ☐   1       2       3       4       5       6       7 |
| --- | --- | --- |
| Were you aware of the PPI plans for the project from the beginning? | | No                  Unsure                   Yes  ☐     ☐     ☐     ☐     ☐     ☐     ☐   1       2       3       4       5       6       7 |
| Was the overall project (not just PPI) explained to you? For example, the aim of the research project. | | No                  Unsure                   Yes  ☐     ☐     ☐     ☐     ☐     ☐     ☐   1       2       3       4       5       6       7 |
| What were your expectations before the project started? | | |
| What were your goals for participating in the project? | | |
| How did you get involved in the study | ☐   Responded to open invitation.  ☐   Through a voluntary organisation  ☐   Through clinicians involved in the study  ☐   Through an established PPI / research centre  ☐   Other (please specify) : ___________ | |
| Is there anything regarding ‘strategy and methods’ you would like to note for future consideration? | | |

**Your Involvement**

| How long did your relationship with the study last? | | No. of months: | |
| --- | --- | --- | --- |
| Select the box that most applies to you. | | ☐  Patient                             ☐  Caregiver  ☐ Public                              ☐  Other: ______ | |
| Which of the following words best describes your role throughout this study? | | ☐  PPI/Public Advisory Committee member  ☐  Steering Committee member  ☐  Embedded Patient Researcher  ☐  Unsure  ☐ Other: _______ | |
| *Select the areas of the project that you were involved in.* | | | *In your opinion, how important was your involvement at each stage of the study?*  Not important                     Not sure                   Very important   1             2              3               4              5              6             7 |
| ☐ | Identification of research topic | | ☐        ☐          ☐          ☐         ☐          ☐        ☐ |
| ☐ | Seeking additional funding | | ☐        ☐          ☐          ☐         ☐          ☐        ☐ |
| ☐ | Design of the research methodology | | ☐        ☐          ☐          ☐         ☐          ☐        ☐ |
| ☐ | Development of the participant information materials | | ☐        ☐          ☐          ☐         ☐          ☐        ☐ |
| ☐ | Provision of training | | ☐        ☐          ☐          ☐         ☐          ☐        ☐ |
| ☐ | Co-researchers in data collection | | ☐        ☐          ☐          ☐         ☐          ☐        ☐ |
| ☐ | Co-researchers in data analysis | | ☐        ☐          ☐          ☐         ☐          ☐        ☐ |
| ☐ | Report writing | | ☐        ☐          ☐          ☐         ☐          ☐        ☐ |
| ☐ | Advisory / steering groups | | ☐        ☐          ☐          ☐         ☐          ☐        ☐ |
| ☐ | Review of reports, lay summaries | | ☐        ☐          ☐          ☐         ☐          ☐        ☐ |
| ☐ | Dissemination Activities | | ☐        ☐          ☐          ☐         ☐          ☐        ☐ |
| ☐ | Development of future research | | ☐        ☐          ☐          ☐         ☐          ☐        ☐ |
| ☐ | Other: _________ | | ☐        ☐          ☐          ☐         ☐          ☐        ☐ |
| Is there anything regarding your involvement that you would like to note for future consideration? | | | |

**Training**

| Did you receive any training for your involvement in the study? | ☐  Yes | ☐  No |
| --- | --- | --- |
| If yes, how was this done? | | |
| Were you introduced to the other team members? | ☐  Yes | ☐  No |
| If yes, how was this done? | | |
| Did you receive any training materials before starting the study? | ☐  Yes | ☐  No |
| If yes, please elaborate. | | |

**Your Experience with the Research Team**

| ‘I felt that I was well prepared to work with researcher partners on this research team.’ | Strongly Agree            Neither            Strongly disagree       ☐     ☐     ☐    ☐     ☐     ☐     ☐            1          2         3        4          5         6          7 | |
| --- | --- | --- |
| ‘I viewed the experience as positive and fulfilling.’ | Strongly Agree            Neither            Strongly disagree       ☐     ☐     ☐    ☐     ☐     ☐     ☐            1          2         3        4          5         6          7 | |
| ‘There was an environment created within the study for respect, trust, and appreciation for mutual learning, other’s realities, relation attributes and strengths and difficulties of team members.’ | Strongly Agree            Neither            Strongly disagree       ☐     ☐     ☐    ☐     ☐     ☐     ☐          1         2          3        4         5          6         7 | |
| Did you ever express any concerns about how you were being treated by other members of the team? | ☐  Yes | ☐  No |
| ‘Enough time was given for you to provide input and deliberation in the process.’ | Strongly Agree            Neither            Strongly disagree       ☐     ☐     ☐    ☐     ☐     ☐     ☐          1         2          3        4         5          6         7 | |
| ‘Enough time was given for you to learn about the project and get to know the other team members prior to beginning the project.’ | Strongly Agree            Neither            Strongly disagree       ☐     ☐     ☐    ☐     ☐     ☐     ☐          1         2          3        4         5          6         7 | |
| If there were problems, how were they handled by the researchers? | | |
| Is there anything regarding your ‘experience with the research team’ that you would like to note for future consideration? | | |

**Adaptability**

| Was feedback provided to researchers by PPI members, in which that feedback influenced decisions and/or initial protocol of the overall research (i.e., like a feedback loop)? | | ☐  Yes | ☐  No |
| --- | --- | --- | --- |
| Has the PPI member role changed from how it was defined from the outset? | | ☐  Yes | ☐  No |
| ‘PPI members contributed to the decisions made in a meaningful and substantive way.’ | Strongly Agree            Neither            Strongly disagree       ☐     ☐     ☐    ☐     ☐     ☐     ☐          1         2          3        4         5          6         7 | | |
| Is there anything regarding ‘adaptability’ you would like to note for future consideration? | | | |

**Communication Methods**

| ‘The way in which the team communicated with me during the project was appropriate.’ | Strongly Agree            Neither            Strongly disagree       ☐     ☐     ☐    ☐     ☐     ☐     ☐          1         2          3        4         5          6         7 |
| --- | --- |
| ‘I felt that I was able to be involved in open dialogue, honest exchange of ideas, conversations about issues, and resolution.’ | Strongly Agree            Neither            Strongly disagree       ☐     ☐     ☐    ☐     ☐     ☐     ☐          1         2          3        4         5          6         7 |
| ‘Flexible communication methods were used to accommodate all participants.’ | Strongly Agree            Neither            Strongly disagree       ☐     ☐     ☐    ☐     ☐     ☐     ☐          1         2          3        4         5          6         7 |
| If so, how was this done? | |
| 'The ‘academic’ language used was translated into plain English, so that all members could understand.’ | Strongly Agree            Neither            Strongly disagree       ☐     ☐     ☐    ☐     ☐     ☐     ☐          1         2          3        4         5          6         7 |
| If so, were you consulted on this ‘translation’? | |
| ‘You were provided with sufficient feedback throughout the study.’ | Strongly Agree            Neither            Strongly disagree       ☐     ☐     ☐    ☐     ☐     ☐     ☐          1         2          3        4         5          6         7 |
| ‘You were asked to provide PPI feedback throughout.’ | Strongly Agree            Neither            Strongly disagree       ☐     ☐     ☐    ☐     ☐     ☐     ☐          1         2          3        4         5          6         7 |
| If so, what methods were used to do this? | |
| Is there anything regarding ‘communication methods’ that you would like to note for future consideration? | |

**Diversity**

| ‘I felt that the PPI member group was representative of the target population.’ | Strongly Agree            Neither            Strongly disagree       ☐     ☐     ☐    ☐     ☐     ☐     ☐          1         2          3        4         5          6         7 |
| --- | --- |
| ‘I felt that the needs of PPI members were considered prior to the project.’ | Strongly Agree            Neither            Strongly disagree       ☐     ☐     ☐    ☐     ☐     ☐     ☐          1         2          3        4         5          6         7 |
| ‘I felt that the team acted to understand cultures and past experiences of members.’ | Strongly Agree            Neither            Strongly disagree       ☐     ☐     ☐    ☐     ☐     ☐     ☐          1         2          3        4         5          6         7 |
| What were the positive impacts of PPI on the research? | |
| What were the negative experiences of PPI on the research, if any? | |
| Is there anything regarding ‘experience and representation’ you would like to note for future consideration? | |

**Compensation**

| ‘Managing expenses posed an issue for my involvement in the study.’ | Strongly Agree            Neither            Strongly disagree       ☐     ☐     ☐    ☐     ☐     ☐     ☐          1         2          3        4         5          6         7 | | |
| --- | --- | --- | --- |
| If yes, could you tell us how? | | | |
| Were you provided compensation for your involvement in the study? | | ☐  Yes | ☐  No |
| If yes, what kind of compensation given? (e.g. gift card, salary, etc) | | | |
| For what activity was this compensation provided? | | | |
| Is there anything regarding ‘compensation’ that you would like to note for future consideration? | | | |

**Boosting Awareness**

| Did you, as a PPI member, co-present the results of the research, or your perspective on the research by being on panels and/or speaking at conferences? | | | ☐  Yes | | ☐  No |
| --- | --- | --- | --- | --- | --- |
| If so, how? | | | | | |
| Were the PPI members asked to use their contacts to send the results of the research to a wider audience? | ☐  Yes | ☐  No | | ☐  Not yet applicable | |
| Did your involvement in this study lead to your collaboration with any other groups (e.g., other institutions, organisations, charities, etc.)? | | | ☐  Yes | | ☐  No |
| If yes, please name them. | | | | | |
| Has your participation led to any changes in your personal and/or professional skills? | | | ☐  Yes | | ☐  No |
| If yes, please specify. | | | | | |
| Were you cited as an author/contributor on any research outputs (e.g. papers, presentations, etc.)? | | | ☐  Yes | | ☐  No |
| Is there anything regarding ‘boosting awareness’ that you would like to note for future consideration? | | | | | |

**Reporting the benefits and challenges of PPI**

| In your opinion, what are some of the unanticipated challenges of PPI in research? |  | |
| --- | --- | --- |
| If your quality-of-life changed as a result of involvement in patient engagement initiatives,  please explain how? |  | |
| Is there anything regarding PPI, overall, that you would like to note for future consideration? |  | |
| ‘I would be likely to participate in PPI research again in the future.’ | | Strongly Agree            Neither            Strongly disagree       ☐     ☐     ☐    ☐     ☐     ☐     ☐          1         2          3        4         5          6         7 |
