# Supplementary material for: Evaluating public and patient involvement in interventional research–A newly developed checklist (EPPIIC)
Source: PLoS One. 2024 Nov 5;19(11):e0301314. doi: 10.1371/journal.pone.0301314 (PMC11537402; doi:10.1371/journal.pone.0301314)
Supplement: S2 Appendix — EPPIIC (Researcher Version). (DOCX) [file pone.0301314.s002.docx]

**S2: Evaluation of PPI for Interventional research Checklist (EPPIIC)**

**EPPIIC (Researcher Version)**

**Policy and Practice**

**Planned Strategy and Methods**

| Was a clear purpose or reason for engagement with PPI identified? | ☐  Yes | ☐  No |
| --- | --- | --- |
| If so, please provide rationale. | | |
| Was there an explicit study design strategy for PPI in the study? | ☐  Yes | ☐  No |
| If yes, please provide a breakdown. | | |
| Did researchers account for PPI time and resources? | ☐  Yes | ☐  No |
| If yes, please provide a breakdown. | | |
| Was there a set budget for remunerating PPI? | ☐  Yes | ☐  No |
| If yes, please provide a breakdown. | | |
| What were the goals, outcomes and impacts of the participation activity? | | |
| How were these planned to be measured? | | |
| How were these measured in reality? | | |
| Is there anything regarding ‘Strategy and Methods’ you would like to note for future consideration? | | |

**Resource Mobilisation**

| ‘There was enough time provided for PPI members to deliberate and contribute input throughout the project.’ | | Strongly Agree          Neither         Strongly disagree          ☐    ☐    ☐    ☐    ☐    ☐    ☐             1        2        3       4        5       6       7 | | |
| --- | --- | --- | --- | --- |
| ‘There was enough time provided for PPI members to learn about the project and get to know the other team members prior to beginning the project.’ | | Strongly Agree         Neither          Strongly disagree          ☐    ☐    ☐    ☐    ☐    ☐    ☐             1        2        3       4        5       6       7 | | |
| ‘There were clearly identified and adequate PPI resources from the outset of the study.’ (e.g., training opportunities, handbooks, relevant aids) | | Strongly Agree         Neither          Strongly disagree          ☐    ☐    ☐    ☐    ☐    ☐    ☐             1        2        3       4        5       6       7 | | |
| ‘There were adequate facilities available within the institution to allow for PPI engagement.’ (e.g., parking, toilets, online access, etc.) | | Strongly Agree         Neither          Strongly disagree          ☐    ☐    ☐    ☐    ☐    ☐    ☐             1        2        3       4        5       6       7 | | |
| Did PPI members receive any training for their involvement in the study? | | ☐  Yes | | ☐  No |
| If yes, please elaborate on the training and/or materials provided. | | | | |
| What percentage of usable funding did this amount represent? (%) | |  | | |
| Identify and rank the 5 main areas of PPI fund usage in terms of amount of money spent. (Largest first)  For example travel expenses, training, remuneration, etc. | *1.*  *2.*  *3.*  *4.*  *5.* | | | |
| Was managing expenses an issue for PPI members? | | ☐  Yes | | ☐  No |
| If yes, what kind of issues were encountered? | | | | |
| What kind of reimbursement was provided for time, travel, and out of pocket expenses? (e.g. gift card / regular salary) | | | | |
| Were any PPI members remunerated or categorised as staff? | ☐  Yes | | ☐  No | |
| Is there anything regarding ‘resources mobilisation’ you would like to note for future consideration? | | | | |

**Reports of PPI**

| Select the areas of the project that involved PPI. | | Report the nature of PPI used at each of the ticked. | | How important was involvement at each stage of the study?  Not imp                                                Very imp  1          2          3           4          5          6         7 |
| --- | --- | --- | --- | --- |
| ☐ | Identification of research topic |  | | ☐     ☐      ☐       ☐      ☐      ☐     ☐ |
| ☐ | Seeking additional funding |  | | ☐     ☐      ☐       ☐      ☐      ☐     ☐ |
| ☐ | Design of the research methodology |  | | ☐     ☐      ☐       ☐      ☐      ☐     ☐ |
| ☐ | Development of the participant information materials |  | | ☐     ☐      ☐       ☐      ☐      ☐     ☐ |
| ☐ | Provision of training |  | | ☐     ☐      ☐       ☐      ☐      ☐     ☐ |
| ☐ | Co-researchers in data collection |  | | ☐     ☐      ☐       ☐      ☐      ☐     ☐ |
| ☐ | Co-researchers in data         analysis |  | | ☐     ☐      ☐       ☐      ☐      ☐     ☐ |
| ☐ | Report writing |  | | ☐     ☐      ☐       ☐      ☐      ☐     ☐ |
| ☐ | Advisory / steering groups |  | | ☐     ☐      ☐       ☐      ☐      ☐     ☐ |
| ☐ | Review of reports, lay        summaries |  | | ☐     ☐      ☐       ☐      ☐      ☐     ☐ |
| ☐ | Dissemination Activities |  | | ☐     ☐      ☐       ☐      ☐      ☐     ☐ |
| ☐ | Development of future research |  | | ☐     ☐      ☐       ☐      ☐      ☐     ☐ |
| ☐ | Other(s) (please specify) |  | | ☐     ☐      ☐       ☐      ☐      ☐     ☐ |
| Is there anything regarding ‘reports of PPI’ you would like to note for future consideration? | | |  | |

**Recruitment**

| How many PPI members were involved in this study? | | Number: |
| --- | --- | --- |
| How did they get involved in the study? | ☐   Responded to open invitation.  ☐   You approached a voluntary organisation  ☐   You approached patients / service users known to the clinicians involved in the study.  ☐   You approached an established PPI group in your research centre  ☐   Other (please specify) : ___________ | |
| Is there anything regarding ‘recruitment’ you would like to note for future consideration? | | |

**Team Engagement**

| ‘You felt you and your team were well prepared to work with PPI on this research.’ | | Strongly Agree         Neither          Strongly disagree         ☐    ☐    ☐    ☐    ☐    ☐    ☐            1        2        3       4        5       6       7 | | | | |
| --- | --- | --- | --- | --- | --- | --- |
| Have the PPI members expressed any concerns about how they were treated by other members of the team, to date? | | | ☐  Yes | | ☐  No | |
| If there were problems, how were they handled? | | | | | | |
| Is there anything regarding ‘Team Engagement’ you would like to note for future consideration? | | | | | | |
| ‘There was evidence of a commitment to understanding community cultures, demographics, and past collaborative experiences of PPI members.’ | Strongly Agree            Neither            Strongly disagree          ☐     ☐     ☐    ☐     ☐     ☐     ☐             1         2         3       4         5         6        7 | | | | | |
| ‘The ability to meet people in settings that are familiar to them has been considered.’ | Strongly Agree            Neither            Strongly disagree          ☐     ☐     ☐    ☐     ☐     ☐     ☐             1         2         3       4         5         6        7 | | | | | |
| Was domain-specific terminology in shared documentation translated into accessible language to facilitate understanding (where feasible/appropriate) and reviewed by PPI members, prior to being shared externally? | | | | ☐  Yes | | ☐  No |
| If applicable, how were PPI member health needs identified? | | | | | | |
| Is there anything regarding ‘gap analysis’ you would like to note for future consideration? | | | | | | |

**Adaptability**

| Was feedback provided to researchers by PPI members, in which that feedback influenced decisions and/or initial protocols of the overall research? (i.e. like a feedback loop) | | ☐  Yes | ☐  No |
| --- | --- | --- | --- |
| Has the PPI member role changed from how it was defined from the outset? | | ☐  Yes | ☐  No |
| If so, how? | | | |
| ‘There was flexibility for respect for experimental knowledge and mutual learning.’ | Strongly Agree            Neither            Strongly disagree          ☐     ☐     ☐    ☐     ☐     ☐     ☐             1         2         3       4         5         6         7 | | |
| ‘An environment was created within the study for respect, trust, and appreciation for other’s realities, relation attributes and strengths and difficulties of team members.’ | Strongly Agree            Neither            Strongly disagree          ☐     ☐     ☐    ☐     ☐     ☐     ☐             1         2         3       4         5         6         7 | | |
| ‘PPI members viewed the experience as positive and fulfilling.’ | Strongly Agree            Neither            Strongly disagree          ☐     ☐     ☐    ☐     ☐     ☐     ☐             1         2         3       4         5         6         7 | | |
| Is there anything regarding ‘adaptability’ you would like to note for future consideration? | | | |

**Experience and Representation**

| Do you have lived experience of the research topic? | ☐  Yes | | ☐  No | ☐ Rather not disclose |
| --- | --- | --- | --- | --- |
| Have you ever participated in research as a PPI member? | | | ☐  Yes | ☐  No |
| Prior to this study, did you have any experience working with PPI in past research? | | | ☐  Yes | ☐  No |
| If so, how was your past experience with PPI? | | | Negative                Neither                Positive      ☐    ☐    ☐    ☐    ☐    ☐    ☐       1        2       3        4       5        6        7 | |
| How did you find your experience with PPI in this research? | | | Negative                Neither                Positive      ☐    ☐    ☐    ☐    ☐    ☐    ☐       1        2       3        4       5        6        7 | |
| What were the inclusion criteria for PPI members? | | | | |
| ‘The PPI member group was representative of the target population.’ | | Strongly Agree            Neither            Strongly disagree          ☐     ☐     ☐    ☐     ☐     ☐     ☐             1         2         3       4         5         6        7 | | |
| ‘There was an effort made to include underserved communities.’ | | Strongly Agree            Neither            Strongly disagree          ☐     ☐     ☐    ☐     ☐     ☐     ☐             1         2         3       4         5         6        7 | | |
| What were the changes in attitude / perception of researchers regarding PPI members? (If any) | | | | |
| What were the positive impacts of PPI on the research? | | | | |
| Were there any negative experiences of PPI relating to the research? | | | | |
| Is there anything regarding ‘experience and representation’ you would like to note for future consideration? | | | | |

**Communication Methods**

| ‘Effective communication strategies have been used throughout the project.’ | Strongly Agree            Neither            Strongly disagree         ☐     ☐     ☐    ☐     ☐     ☐     ☐            1         2         3       4         5         6        7 |
| --- | --- |
| ‘Communication strategies allowed for open dialogue, honest exchange of ideas, conversations about issues, and resolution.’ | Strongly Agree            Neither            Strongly disagree          ☐     ☐     ☐    ☐     ☐     ☐     ☐             1         2         3       4         5         6        7 |
| ‘Flexible communication methods were used to accommodate all participants.’ | Strongly Agree            Neither            Strongly disagree          ☐     ☐     ☐    ☐     ☐     ☐     ☐             1         2         3       4         5         6        7 |
| If yes, how was this done? | |
| Is there anything regarding ‘communication methods’ you would like to note for future consideration? | |

**Management, and Implementation of PPI Recommendations**

| ‘PPI members contributed to relevant decisions in a meaningful and substantive way.’ | | Strongly Agree            Neither            Strongly disagree          ☐     ☐     ☐    ☐     ☐     ☐     ☐           1         2         3       4         5         6        7 | |
| --- | --- | --- | --- |
| *Provide all instances where PPI members’ input influenced decisions.* | *Importance of task* | | *Impact of PPI involvement* |
|  | ☐  Small  ☐  Medium  ☐  Large | | ☐  None  ☐  Low  ☐  Moderate  ☐  High |
|  | ☐  Small  ☐  Medium  ☐  Large | | ☐  None  ☐  Low  ☐  Moderate  ☐  High |
|  | ☐  Small  ☐  Medium  ☐  Large | | ☐  None  ☐  Low  ☐  Moderate  ☐  High |
|  | ☐  Small  ☐  Medium  ☐  Large | | ☐  None  ☐  Low  ☐  Moderate  ☐  High |
|  | ☐  Small  ☐  Medium  ☐  Large | | ☐  None  ☐  Low  ☐  Moderate  ☐  High |
|  | ☐  Small  ☐  Medium  ☐  Large | | ☐  None  ☐  Low  ☐  Moderate  ☐  High |
|  | ☐  Small  ☐  Medium  ☐  Large | | ☐  None  ☐  Low  ☐  Moderate  ☐  High |
|  | ☐  Small  ☐  Medium  ☐  Large | | ☐  None  ☐  Low  ☐  Moderate  ☐  High |
| Is there anything regarding ‘management and implementation of PPI recommendations’ you would like to note for future consideration? | | | |

**Participatory Culture**

**Boosting Awareness**

| Did (or will) PPI member(s) co-present the results of the research, or their perspective on the research by being on panels, co-authoring papers and/or speaking at conferences? | | ☐  Yes | ☐  No |
| --- | --- | --- | --- |
| If so, how? | | | |
| Were the PPI members asked to use their contacts to send the results of the research to a wider audience? | | ☐  Yes | ☐  No |
| Did PPI lead to collaboration with any other groups?  (e.g., other institutions, organisations, charities, etc.) | | ☐  Yes | ☐  No |
| If so, how? | | | |
| What number of PPI members were cited as authors/contributors in this research? | Number: | | |
| Is there anything regarding ‘boosting awareness’ you would like to note for future consideration? | | | |

**Participatory Feedback**

| Did PPI members have the opportunity to provide feedback on their participation? | | ☐  Yes | ☐  No |
| --- | --- | --- | --- |
| How was this carried out? | ☐ Interview                      ☐  Open-ended evaluation form     ☐ Survey                           ☐  Other:_____ | | |
| ‘I would be likely to involve PPI in future research.’ | | Strongly Agree            Neither            Strongly disagree          ☐     ☐     ☐    ☐     ☐     ☐     ☐             1         2         3       4         5         6        7 | |
| Is there anything regarding ‘participatory feedback’ you would like to note for future consideration? | | | |

**Influencing Outcomes of PPI**

| In your opinion, what are some of the unanticipated challenges of PPI in research, if any? |
| --- |
| Describe the influence of any process or contextual factors, that enabled or hindered the impact of PPI, if any. |
| Describe any conceptual or theoretical development in PPI that has emerged, if any. |
| Is there anything regarding ‘outcomes of PPI’ you would like to note for future consideration? |
