# Supplementary material for: Evaluating public and patient involvement in interventional research–A newly developed checklist (EPPIIC)
Source: PLoS One. 2024 Nov 5;19(11):e0301314. doi: 10.1371/journal.pone.0301314 (PMC11537402; doi:10.1371/journal.pone.0301314)
Supplement: S3 Appendix — (DOCX) [file pone.0301314.s003.docx]

**Supplementary File 3**

### Application of the EPPIC to the COB-MS feasibility trial

Following its completion, the checklist was piloted on the COB-MS trial (1;2;3). Specifically, the trial employed a PPI member as a contracted researcher [becoming an *embedded patient researcher* (EPR; 4)], included two PPI members in the Trial Steering Committee and created an external PPI consultation group.

The EPPIIC (PPI member form) was completed by the trial’s EPR, while the researcher form was completed by trial PI. Overall, the checklists identified areas of strengths in terms of PPI inclusion and highlighted areas for future improvement. The results of the checklist’s application are presented under the three themes of the EPPIIC. Direct quotes from the checklists are presented for further context. These results are summarised in Tables 1 and 2 and 3, with further description of presented in-text below. The checklist asked both cohorts to provide their opinions to statements through a Likert ‘significance’ or ‘agreement’ scale. The box that was chosen by the respondent reflected the strength of their opinion towards the statement provided in each section.

### Policy and Practice

In the Policy and Practice section, both checklists reported that the design strategy and reason for PPI was clearly defined prior to the beginning of the trial. Both PPI member and researcher believed the goal of PPI was to improve the quality of research, and therefore, improve the quality of life of patients.

*“The goal was to include PPI members as research partners throughout the entire research process. We wanted to do this in order to improve the quality, relevance, and acceptability of the research for people with MS”* – Researcher form.

*“To improve the quality of the research, which would ultimately improve the quality of life of people living with MS, in particular their cognition.”* – PPI member form

In terms of resources, both agreed that sufficient time was given to learn about the project and for PPI members to provide input and deliberation in the process. Each form found that training materials were provided, however, the PPI form added that while they were given a manual, *“there was no training on how to perform my role”* (PPI member form).

The researcher and PPI member survey concluded that managing expenses was an issue for PPI members. They were compensated for their contribution by gift card, salary or one-off payment directly into their bank account. The researchers found difficulty with timely distribution of compensation (though this may have been more so an artefact of institutional finance procedures than a reflection on researcher motivations).

In total, six PPI members were involved and were recruited through engaging a voluntary organisation relevant to the trial’s focus (i.e. multiple sclerosis). While both teams found that this working environment created trust and respect for individual realities and mutual learning, the PPI team member was unsure if they initially felt prepared to work with the research team. There was mutual agreement that sufficient time for gaining familiarity with the trial and for input and deliberation was granted. The research team stated they identified health needs and catered for them by allowing accessible facilities for in-person meetings; however, the PPI member team found these facilities unsuitable for their needs.

*“When meeting took place in person, we ensured parking was available nearby and accessible buildings were used. Meetings were held at a time that suited PPI members.” –* Researcher form

*“The university found it very difficult to find a place for me to store a mobility scooter, and the eventual location was not suitable. The disabled toilets were not solely for disabled people, but for everyone, which defeats their purpose. The university did not provide me with equipment supports, which I had to provide from my own resources.”* – PPI member form

There were reciprocal views that the language used within intervention materials was translated into ‘plain English’ from academic language. The PPI checklist expressed that without experience or background in teamwork, PPI members may struggle to perform their role.

*“I had experience in my previous work life of working in groups, and I also had experienced what research is like when I was doing my degree. This made the role easier for me, however, if I didn’t have this prior knowledge, it would have been more difficult to perform the role.” –* PPI member form

There was mutual agreement that the participants represented the target population; however, the research team did not make an extra effort to include underserved communities (e.g. given that underserved communities were not the target focus of the research, rather those with multiple sclerosis). Both surveys found that communication methods were adequate and allowed for honest conversations to support new ideas and decisions. The two forms also agreed that these methods were flexible as engagement was forced online due to the COVID-19 pandemic. PPI member involvement allowed contribution and implementation to decision-making. Table 1, below, summarises the main points from this theme from the perspective of the researcher and the PPI member.

**Table 1: Summary of results regarding Policy and Practice**

| **Policy and Practice** | |
| --- | --- |
| **Researcher** | **PPI Member** |
| **Planned Strategy & Methods** | |
| - The purpose of engagement was to shape study design, establish networks, dissemination of materials, providing support/ advice; and to enhance the quality and acceptability of the research for people with MS. - PPI strategy was planned so partnership involved recruitment documentation, study requirements and burden for participants discussion, content of acceptability interviews, dissemination of findings and experiences. - Embedded patient researcher was hired and predicted compensation breakdown was planned. This included budget for travel, subsistence, and time. | - The reason for PPI and overall project was explained to PPI members. - PPI members were unsure of the PPI plans for the project at the beginning. - PPI members expected the researchers to show things connected with the trial, such as documents, manuals, to see if they were suitable for people living with multiple sclerosis. - Goals were to improve quality of research, to improve the quality of life of people living with MS. |
| **Resource Mobilisation** | |
| - Agreed that there was enough time provided for PPI members to learn about the project, and for input and deliberation. - There were clearly identified and adequate PPI resources and facilities within the institution for engagement. - PPI members received participation information sheet, participant manual, and COB-MS training videos as training materials. - 5.8% of usable funding was spent solely on PPI efforts, with main areas including EPR salary, travel costs, PPI consultation fees, COVID-19 related meetings. - Managing expenses was an issue for PPI members, and there were challenges with distribution of compensation in a timely manner. - PPI members were categorised as staff and reimbursement was given in the form of Gift cards, salary, and bank transfers. | - Agreed that there was enough time provided for PPI members to learn about the project, and for input and deliberation. - Training was provided on an event basis. EPR was asked to prepare a Patient Information Sheet. EPR was provided with a manual about the project but no resources on how to perform their role. - PPI members were introduced to the other team members face-to-face, and online during the COVID-19 pandemic. - Managing expenses was an issue for PPI members, in that the reimbursement was often insufficient to cover travel, sustenance and time spent. - Compensation was provided in the form of a salary for the role of EPR. |
| **Reports of PPI** | |
| - PPI members were involved in all areas of research, apart from as co-researchers in data analysis. - Involvement in every other stage was considered “very important”, except in ‘identification of Research Topic’. | - EPR relationship with the trial lasted 36months. - They considered all areas of the trial that they were involved in important, except the “Identification of the research topic”. They were unsure of the significance of PPI member involvement in the steering groups (to note the EPR was not a member of this group). |
| **Recruitment** | |
| - Six PPI members were recruited in during the trial, by researchers approaching the patient organisation (MS Ireland). | - EPR was recruited to the trial through an advertisement in the MS Society of Ireland Research Newsletter. |
| **Team Engagement** | |
| - Researchers felt themselves and the team were well prepared to work with PPI, and that no concerns were expressed by PPI members. - There was a commitment to understanding community cultures and past experiences of PPI members. - The ability to meet people in a setting familiar to them was considered, and language was translated into accessible language to facilitate understanding. - Health needs were identified as in-person meetings were held in accessible facilities with nearby parking, and at a time that suited PPI members. - During the COVID-19 pandemic, phone stands were given to participants to facilitate online engagement. | - EPR was unsure if all PPI members felt prepared to work with researchers. - There was an environment for trust, respect and mutual learning, and the team acted to understand cultures and past experiences of members. - They did express concerns over their treatment by the team; however the issue was resolved by speaking with the PI of the team. - Academic language was translated into plain English, PPI members were not consulted for this translation, however, the research team explained any terms necessary. - The EPR expressed that they had previous experience of working in a team, however, felt that anyone without this knowledge might struggle to preform that role. |
| **Adaptability** | |
| - A feedback loop was used to communicate. - The PPI role changed from PPI member to embedded patient researcher. - Tasks were developed as the trial continued. - There was flexibility and an environment for experimental knowledge, trust, and respect. - PPI members viewed the experience as positive and fulfilling. | - A feedback loop was used to communicate. - The PPI member role changed from how it was defined from the outset. - PPI members viewed the experience as positive and fulfilling. |
| **Experience & Representation** | |
| - The researcher team has no lived experience of the topic. - They had previous experience of working with PPI but viewed that experience somewhat negatively. - In contrast, they viewed this experience as highly positive. - The was a slight agreement that the six PPI member-group was a representative of the target population; however, it was disagreed that there had been an effort to include underserved communities. | - The PPI members felt that their group was representative of the target population, and slightly agreed that the needs of the PPI members were considered prior to the project. |
| **Communication Methods** | |
| - Communication strategies were used that allowed for open dialogue, honest exchange of ideas, and conversation regarding issues. - It was slightly agreed that flexible communication methods were used to accommodate all participants, using email, phone and in-person meetings. - COVID-19 pandemic forced a change engagement to online interaction. | - The team communicated to PPI members in an appropriate manner that allowed for open dialogue, honest exchange of ideas and conversations regarding issues. - It was strongly agreed that flexible communication methods were used to accommodate all participants, through online engagement, however, without the aid of the institution. |
| **Management, & Implementation of PPI Recommendations** | |
| - PPI members contributed to relevant decisions in a meaningful and substantive way. | - PPI members contributed to decisions in a meaningful and substantive way. |

### Participatory Culture

The second thematic section of the EPPIIC reported that PPI inclusion in this trial led to further collaborations between the research team and PPI members (e.g.in joint presentations and articles not directly related to the COB-MS). Reports were also provided on PPI members co-presenting results of the research and their perspectives to different audiences. One PPI member, to date, was cited as a co-author, while the PPI group have been acknowledged on multiple publications. Involvement in research facilitated the PPI members to gain knowledge on how PPI can influence the quality of research.

*“I am more knowledgeable of the research process and know how the patient can help improve the quality of research.” –* PPI member form

Further results are presented from this section in Table 2.

**Table 2 - Summary of results regarding Participatory Culture**

| **Participatory Culture** | |
| --- | --- |
| **Researcher** | **PPI Member** |
| **Boosting Awareness** | |
| - PPI members co-presented the results of research / their perspective by writing a blog posts, providing keynote speeches, presenting oral and poster presentations to lay and scientific audiences. - PPI members were not asked to use their personal contacts to send the results to a wider audience. - PPI led to collaboration with other national and international groups. - One PPI member (to date) named as a co-author on publications, while the PPI group are acknowledged on multiple publications. | - The EPR co-presented the results of the research, or their perspective at many functions, in many different formats, and to many different audiences. - The trial also led to collaboration with national and international groups. - Personal and/or professional skills changed in that the EPR found they became more knowledgeable of the research process and how patients can improve the quality of research. - The EPR was cited as an author / contributor on many COB-MS research outputs. |
| **Participatory Feedback** | |
| - The EPR had the opportunity to provide feedback on their participation through this checklist. However, feedback was not sought from the wider PPI groups as it was not included in the ethics application. | - Sufficient PPI feedback was provided to PPI members throughout the trial. - PPI members were asked to provide feedback throughout. This was done at weekly meetings. |

### Influencing the Outcomes of PPI

The final section of the EPPIIC focused on the strengths of and challenges faced by PPI in the COB-MS trial. Specific examples included challenges in organisation of meetings when PPI were not responsible for chairing them, as well as wider organisational constraints such as the institution’s processes around payment (see Table 3). The PPI member reported challenges with the facilities of the institution but found that a clear sense of purpose was gained from their involvement in the study.

*“I was able to use my experience of something negative, chronic illness, for something positive. It gave me a purpose again.” –* PPI member form

Conceptual developments that emerged concerned the creation of the role of ‘*Embedded Patient Researcher*’. This role was defined as it involved a greater depth of involvement in comparison to the PPI advisory panel. This was the first time this had been done in this institution. The research team also identified ‘thorough evaluation’ as an important consideration for future PPI inclusion.

**Table 3: Summary of notes and ideas regarding Influencing the Outcomes of PPI**

| **Influencing the Outcomes of PPI** | |
| --- | --- |
| **Researcher** | **PPI Member** |
| - Challenges of PPI in this trial included inclusion of PPI members when meetings were not chaired by the research team, and retention of PPI members during the COVID-19 pandemic due to unfamiliarity with Zoom/online meetings. - The university’s process surrounding payment hindered the reputation of PPI in the trial. - Motivation from the PPI panel and EPR enabled the impact of PPI. - Having an EPR made the wider PPI tasks more successful and easier to manage. - The term ‘*Embedded Patient Researcher*’ was coined. - The researcher would be likely to involve PPI in future research. - Positive impacts on PPI in research included recruitment, developing patient materials, integration, engagement with trial participants, communication & dissemination plan, and collaboration. | - Challenges of PPI inclusion: 1) the university facilities were often not appropriate for the PPI members; and 2) did not provide any equipment resources. - PPI member quality of life changed as their involvement allowed them to create a positive outcome from a negative situation and helped them find purpose. - In the future, a buddy system could be proposed to involve inexperienced PPI members. - Currently the social welfare system in Ireland does not permit people in receipt of some payments to engage in PPI research. - The EPR would be likely to participate in PPI research again. - Positive impacts of PPI on the research were the patient information sheet, participant manual, phone stand, newsletter, etc. - No negative experiences of PPI on research were identified. |

### Discussion PPI in the COB-MS using the EPPIIC

The application of the EPPIIC to the COB-MS feasibility trial provided insight into PPI factors that were beneficial and those that were challenging. The ‘Policy and Practice’ theme revealed that there was general agreement over the reasoning for including PPI in the research and the development of an environment of respect and trust. There was slight disparity within the suitability of facilities used and training resources mobilised. A good relationship between PPI members and researchers was revealed, with any conflict being found to be constructive and yielding an acceptable solution. There was a pre-planned structure to the inclusion and engagement of PPI within COB-MS. A review assessing the impact of PPI within MS specific trials found that while many different approaches may be taken, inclusion in ‘trial design’ was crucial to giving PPI members power to impact the research and various communication channels are required to enhance participation, as utilised in the COB-MS (5). Other consistent findings between the current research and Gray and colleagues’ review (5) were included the attitude of PPI members towards research, as well as the importance and methods of reimbursement. The benefits of PPI organisation and practice were reflected in the decisions that led to higher-quality research, as a direct result of PPI.

The theme of ‘Participatory culture’ showed a ‘fully intertwined’ partnership as PPI members were involved in citation and publication. The impact of PPI in this trial also led to further collaborations for the PPI members and researchers. The team sought feedback from the PPI team to allow them to express any concerns. This shows a true effort to involve the PPI team as fellow researchers and reflected a good relationship between the researcher and PPI member team.

The assessment of outcome-based metrics was included within the ‘Influencing the outcomes of PPI’ section. This again reflected that while accommodations were made for PPI involvement, the institution did not seem entirely suitable for PPI members to access. For example, a lack of equipment supports or facilities to store mobility aids. However, this was overcome, to some extent, by the adaptation of online meetings during the COVID-19 pandemic. This theme also exhibited insight into the effect of involvement for PPI members, as they found purpose in increasing the quality of research – feeling the ability to turn their negative experiences into positive input into possible clinical intervention. Positive impacts that PPI brought further included recruitment, proper engagement with participants, developing patient materials and communication and collaboration. Both parties agreed that they would get involved in PPI research in the future, reflecting a positive outlook on their own involvement.

References:

1. Dwyer, C.P., Alvarez-Iglesias, A., Joyce, R., Counihan, T. J., Casey, D. & Hynes, S.M. (2020). Evaluating the feasibility and preliminary efficacy of a Cognitive Occupation-Based programme for people with Multiple Sclerosis (COB-MS): protocol for a feasibility cluster-randomised controlled trial. Trials, 21(1), 269. https://doi.org/10.1186/s13063-020-4179-5
2. Dwyer, C.P., Alvarez-Iglesias, A., Joyce, R. Counihan, T. J., Casey, D. & Hynes, S.M. (2023). Evaluating the feasibility and preliminary efficacy of a Cognitive Occupation-Based programme for people with Multiple Sclerosis (COB-MS): an update to the protocol for a feasibility cluster-randomised controlled trial. Trials 24, 48. <https://doi.org/10.1186/s13063-023-07080-y>
3. Hynes SM, Dwyer CP, Alvarez-Iglesias A, Rogers F, Joyce RA, Oglesby MH, Moses A, Bane E, Counihan TJ, Charamba B. A cluster-randomised controlled feasibility trial evaluating the Cognitive Occupation-Based programme for people with Multiple Sclerosis (COB-MS). Neurological Sciences. 2024 Sep 24:1-8.
4. Joyce R, Dwyer CP & Hynes S. M. (2021). Twelve months into a feasibility trial: reflections on three experiences of public and patient involvement in research [version 2; peer review: 3 approved]. HRB Open Research, 4:11 <https://doi.org/10.12688/hrbopenres.13205.2>
5. Gray E, Amjad A, Robertson J, Beveridge J, Scott S, Peryer G, et al. Enhancing involvement of people with multiple sclerosis in clinical trial design. Mult Scler. 2023;29(9):1162-73.
